# Molecular Characterization of Patients with Cryptorchidism: Preliminary Search for an Expression Profile Related to That of Testicular Germ-Cell Tumors

**DOI:** 10.3390/diagnostics13183020

**Published:** 2023-09-21

**Authors:** Fabiola García-Andrade, Rosa María Vigueras-Villaseñor, Margarita Dolores Chávez-Saldaña, Julio César Rojas-Castañeda, Ivan Uriel Bahena-Ocampo, Elena Aréchaga-Ocampo, Mauricio Flores-Fortis, José Díaz-Chávez, Luis Alonso Herrera, Daniel Adrian Landero-Huerta

**Affiliations:** 1 Laboratorio de Biología de la Reproducción, Instituto Nacional de Pediatría, Ciudad de Mexico 04530, Mexico; 2 Posgrado en Biología Experimental, Universidad Autónoma Metropolitana, Unidad Iztapalapa, Ciudad de Mexico 09310, Mexico; 3 Departamento de Ciencias de la Salud, Universidad Autónoma Metropolitana, Unidad Iztapalapa, Ciudad de Mexico 09310, Mexico; 4 Departamento de Ciencias Naturales, Universidad Autónoma Metropolitana, Unidad Cuajimalpa, Ciudad de Mexico 05348, Mexico; 5 Posgrado en Ciencias Naturales e Ingeniería, Universidad Autónoma Metropolitana, Unidad Cuajimalpa, Ciudad de Mexico 05348, Mexico; 6 Unidad de Investigación en Cáncer, Instituto de Investigaciones Biomédicas-Universidad Nacional Autónoma de México, Instituto Nacional de Cancerología, Ciudad de Mexico 14080, Mexico; 7 Escuela de Medicina y Ciencias de la Salud-Tecnológico de Monterrey, Ciudad de Mexico 14380, Mexico

**Keywords:** cryptorchidism, hsa-miR-371-373 cluster, hsa-miR-367, *IGF1R*, *LATS2*, testicular germ-cell tumor

## Abstract

Cryptorchidism (CO) is a risk factor for the development of testicular germ-cell tumors (TGCT). This is supported by reports showing the persistence of gonocytes in CO patients. These cells are proposed to be related to the development of germ-cell neoplasia in situ (GCNIS), which is considered the precursor stage/lesion of TGCT. Therefore, it is proposed that some patients with CO could express some molecular markers related to TGCT. In this study, we analyzed testicular tissue samples from CO, TGCT, and controls. We determined the expression of POU5F1, PLAP, and KIT by immunohistochemistry and that of the hsa-miR-371-373 cluster, hsa-miR-367, and *LATS2*, *PTEN*, and *IGFR1* genes by RT-qPCR. We then carried out a bioinformatic analysis to identify other possible candidate genes as tumor biomarkers. We found that 16.7% (2/12) of the CO patients presented increased expression of POU5F1, KIT, PLAP, hsa-miR-371-373, and hsa-miR-367 and decreased expression of *LATS2* and *IGF1R*. Finally, the genes *ARID4B*, *GALNT3*, and *KPNA6* were identified as other possible candidate tumor biomarkers. This is the first report describing the expression of the hsa-miR-371-373 cluster, hsa-miR-367, *LATS2*, and *IGF1R* in the testicular tissues of two CO patients with cells immune-positive to POU5F1, PLAP, and KIT, which is similar to what is observed in TGCT.

## 1. Introduction

Cryptorchidism (CO; OMIM # 219050), or undescended testis, is the most common genitourinary anomaly in live male newborns and affects one or both testicles; this malformation is classified according to the anatomical location of the testicle, such as abdominal or inguinal. It can occur in association with various chromosomal syndromes or in isolation (non-syndromic). It is considered a multifactorial condition in which environmental, lifestyle, hormonal, and genetic factors are involved [1]. This malformation increases the risk of developing testicular germ-cell tumors (TGCT; OMIM # 273300) [2].

Germ-cell neoplasia in situ (GCNIS) is a precursor stage/lesion from which invasive TGCTs are derived [3]. It was suggested that GCNIS originates from gonocytes during embryonic development, as suggested by the morphological similarities between gonocytes and atypical cells present in the lesion [4], as well as by their expression of proteins such as POU domain class 5 transcription factor 1 (POU5F1), the transcription factor AP2γ (AP2γ), the tyrosine kinase receptor c-Kit (KIT), placental-like alkaline phosphatase (PLAP), sal-like protein 4 (SALL4), the homebox transcription factor Nanog (NANOG), and thy-1 cell surface antigen (THY1) [5,6]. Some reports show the persistence of gonocytes in pediatric CO patients [7], which did not mature into type A spermatogonia [8]. It was reported that a low proportion of CO patients older than one year of age may present the expression of at least one and up to five previously described proteins. Therefore, it is believed that the persistence of undifferentiated gonocytes with pluripotent capacity beyond the differentiation period could be responsible for the development of malignancy in some of these CO patients [6,8]. In addition, it was shown that fetal gonocytes without any morphological alteration obtained from abortions express the hsa-miR-371-373 and hsa-miR-302-367 clusters in a similar way to GCNIS tissue and serum samples from GCNIS adult patients. [9,10]. However, even when there is a clinical relationship between CO and TGCT, the molecular pathways that support the association between the two conditions are unknown [11].

The expression of some microRNAs (miRNAs) in TGCT has also been reported [12,13]. In particular, overexpression of the hsa-miR-371-373 cluster and of hsa-miR-367 was identified regardless of tumor histological subtype or anatomical site or of the patient’s age [14]. To date, only overexpression of these miRNAs has been reported in TGCT, which increases their potential as biomarkers for the future in the clinical practice [15].

Likewise, in TGCT, the sub-expression of multiple essential genes in various biological pathways necessary for the maintenance of the tumor phenotype has been reported [16], indicating them as possible targets of the hsa-miR-371-373 cluster and of hsa-miR-367. Therefore, in this work, we performed molecular characterization of testicular tissues from CO patients on the basis of the expression profile of TGCT.

## 2. Materials and Methods

### 2.1. Patients

We analyzed 36 paraffin-embedded testicular tissue samples (from 36 patients) distributed in 3 different study groups: (1) a control group from the Instituto de Ciencias Forenses (INCIFO, Mexico City, Mexico), (2) a CO group, and (3) a TGCT group, all with 12 samples, from the pathology service of the Instituto Nacional de Cancerología (INCan, Mexico City, Mexico). Prior to the selection of the tissues, we verified in the patients’ clinical history that the patients had, at minimum, Mexican ancestry for at least two generations, with a 46,XY karyotype and no disorders of sex development.

### 2.2. Histological and Immunohistochemical Analysis

Paraffin-embedded testicular tissues were sectioned on a microtome (Leica RM 2155; Microsystems, Nussloch Gmbh, Heidelberger, Germany), producing 4 µm thick slices that were mounted on poly-L-lysine-coated slides (Sigma-Aldrich, St. Louis, MO, USA). The tissue sections were deparaffinized and hydrated in a graded ethanol series. For each patient, one slide containing the tissue sections was stained with hematoxylin and eosin, and another was mounted on a drop-cover plate (Thermo Scientific, Waltham, MA, USA). The slides were incubated in 0.1 M EDTA pH 9 (immuno Dual retrieval with EDTA) for POU5F1 staining and in 0.1 M citrate buffer pH 6.0 (Bio SB, Sta. Barbara, CA, USA) for vimentin, KIT, and PLAP staining and placed in a microwave inside a pressure cooker (NordicWare^®^ Microwave Tender Cooker, Biogenex, San Ramon, CA, USA) for 1 min at high power (1000 W). After cooling, the tissue sections were incubated in 0.9% H_2_O_2_ in distilled water for 5 min. The sections were consecutively incubated with an anti-vimentin mouse monoclonal antibody at a dilution of 1:50 (Dako Cytomation, Carpinteria, CA, USA, M 0725, lot 092) for 45 min at room temperature, followed by a secondary antibody coupled to biotin, and subsequently were treated with a solution of horseradish peroxidase coupled to streptavidin for 30 min at room temperature. The diaminobenzidine reaction was used to reveal tissue immunoreactivity. For POU5F1 staining, a mouse monoclonal antibody was used at a 1:50 dilution (Santa Cruz, Biotechnology, Dallas, TX, USA, SC 5279, lot F2513), incubating the samples for 45 min at room temperature, followed by treatment with a biotin-free system (secondary antibody goat anti-mouse-HRP and polymer (MACH 2 Mouse HRP-Polymer, Biocare Medicals, Concord, CA, USA, MHRP520 L)) for 30 min at room temperature, according to the manufacturer’s instructions. The reaction was evidenced using diaminobenzidine (BSB 0005, Bio SB, Santa Barbara, CA, USA).

For KIT/CD117 staining, a mouse monoclonal antibody (Leica Novocastra Bannock, Burn, IL, USA, L-CD117, lot 6014169) was applied at a 1:50 dilution for 45 min at room temperature, followed by incubation with a biotin-coupled secondary antibody and a streptavidin-coupled horseradish peroxidase solution for 30 min at room temperature; immunoreactivity was finally revealed using a diaminobenzidine substrate mix (Bio SB). For PLAP staining, a 1:50 diluted mouse monoclonal antibody (Dako Cytomation M7191) was applied for 45 min at room temperature, followed by incubation with a secondary antibody coupled to biotin and with streptavidin coupled to alkaline phosphatase (BioGenex, Fremont, CA, USA, LA000-UL) in the presence of an alkaline phosphatase substrate for 30 min (BioGenex), according to the manufacturer’s instructions. At the end of the reaction, the sections were counterstained with Gill’s hematoxylin and covered with entellan medium (Merck, Darmstadt, Germany).

Vimentin staining was performed to verify the integrity of the antigenic sites. Negative-control sections were processed identically, but the primary antibody incubation step was omitted. None of the negative controls showed immunoreactivity. Tumor tissue was included as a positive control. For the identification of germ cells showing a positive signal, 5 to 10 cross-sections of seminiferous tubules were examined. The samples showing focal staining in germ cells in some seminiferous tubules were classified as positive.

### 2.3. RNA Isolation and RT-qPCR

miRNAs and total RNA were extracted using the Qiagen^®^ miRNeasy FFPE Kit, Hilden, Germany, and the FFPE RNA/DNA Purification Plus Kit (Cat. 54300), Norgen Biotek Corp., Thorold, Ontario, Canada, respectively. Reverse transcription of the hsa-miR-371-373 cluster and hsa-miR-367 was performed using the TaqMan^®^ MicroRNA Reverse Transcription Kit, Waltham, MA, USA (Applied Biosystems). On the other hand, for mRNA analysis of the large tumor suppressor kinase (*LATS2*), phosphatase and tensin homolog (*PTEN*), and insulin-like growth factor 1 receptor (*IGF1R*) genes, the TaqMan^®^ Reverse Transcription Reagents Kit N8080234 (Applied Biosystems) was used. In both cases, we obtained cDNA from aliquots of 100 ng of RNA for each assay. Subsequently, qPCR assays were performed in triplicate in an Applied Biosystem StepOne^TM^ thermocycler, Foster city, CA, USA, starting with 2 µL of cDNA and using the Taqman Universal PCR Master Mix. The expression levels were determined using the 2^−ΔΔCt^ method. For data normalization, we determined the expression of small nucleolar RNA, C/D box 44 (RNU44), and U6B small nuclear RNA (RNU6B) for the miRNAs, and that of human glyceraldehyde-3-phosphate dehydrogenase (*GAPDH*) for the mRNAs, as endogenous controls. The details of the probes used and the assay conditions are reported in Supplementary Figure S1.

### 2.4. Selection of Candidate Genes

Genes reported in the literature as validated targets in TGCT and some other pathologies were considered. We also evaluated the failing of 3′-untranslated-region (3′UTR) recognition of the gene by the seed region of at least one of the miRNAs analyzed by using the microRNA target prediction database (miRDB) [17] and TargetScanHuman [18]. Then, for the selected genes, differential expression graphs between TGCT and control samples, as well as between the different histological types and stages of TGCT, were obtained from the Gene Expression Profiling Interactive Analysis (GEPIA2) web server [19] and the University of Alabama at Birmingham Cancer Data Analysis (UALCAN) Portal [20].

### 2.5. Bioinformatic Analysis

The gene expression datasets GSE65026 and GSE25518, annotated in the Array Express platform from the microarray Affymetrix Human Genome U133 Plus 2.0, were reviewed. The datasets included control tissues and TGCT gene expression data from pure seminoma, teratoma, embryonal carcinoma, and yolk sac tumor cell lines. These data were processed using the Transcriptome Analysis Console (TAC) software, version 4.0, of Applied Biosystems™, in order to obtain the profiles of differentially expressed transcripts (DETs) based on a fold change of <−2 or >2. At all times, values of *p* ≤ 0.05 were considered as indicating significant results. From this profile, we selected the possible common transcripts downregulated by the 4 miRNAs in TGCT. For this, we compared our results with the data annotated in miRtarbase [17] and TargetScanHuman [18]. Then, this last profile was submitted to functional enrichment analysis with Gene Ontology (GO) [21] and the Kyoto Encyclopedia of Genes and Genomes (KEGG) [22] using the R package (version 1.22.0). If there were more than 10 GO annotation and pathway enrichments, only the top 10 terms with adj. *p* < 0.05 were extracted. Finally, differential expression graphs for TGCT and control samples, as well as for the different histological types and stages of TGCT from GEPIA2 [19] and UALCAN [20], were obtained, in order to select other possible candidate genes.

### 2.6. Statistical Analysis

The normal distribution of the expression data between groups was verified using the Shapiro–Wilk test. Following this, a comparison was performed using a nonparametric median comparison test, and the size of the effect was expressed through the d value and its corresponding confidence intervals at 95% in IBM SPSS Statistics for Windows, version 21.0. (IBM Corp., Armonk, NY, USA), and GraphPad Prism 8 was used to represent the data in box-and-whisker plots. At all times, values of *p* ≤ 0.05 were considered significant.

## 3. Results

### 3.1. Histological and Immunohistochemical Analysis

The clinical and/or pathological characteristics of the samples, as well as the histological interpretation of the data, are shown in Table 1. For the control group, we identified a normal histology (cell types according to age) in the samples from infant-age individuals; the samples from pubertal individuals and/or adults presented normal spermatogenesis, except for one of them which showed hypo-spermatogenesis (all germ cell stages present, including spermatozoa, but we observed a clear decline in the number of germ cells), as shown in Table 1A. For the CO group, four samples presented normal histology according to patient age, seven presented tubular atrophy (smaller seminiferous tubes with few cells inside), and only one presented maturation arrest (incomplete spermatogenesis, not beyond the spermatocyte stage), as shown in Table 1B. Regarding the TGCT group, histological alterations typical of testicular neoplasia were identified (large, poorly differentiated, multinucleated cells, with prominent nucleoli, clear cytoplasm, lesions in the adjacent parenchyma, poorly defined lobules, necrosis, and in the case of teratoma, mature or immature cells with components of different types of tissue), evidencing various pure and mixed tumor histological types, as shown in Table 1C.

**Figure 1:**
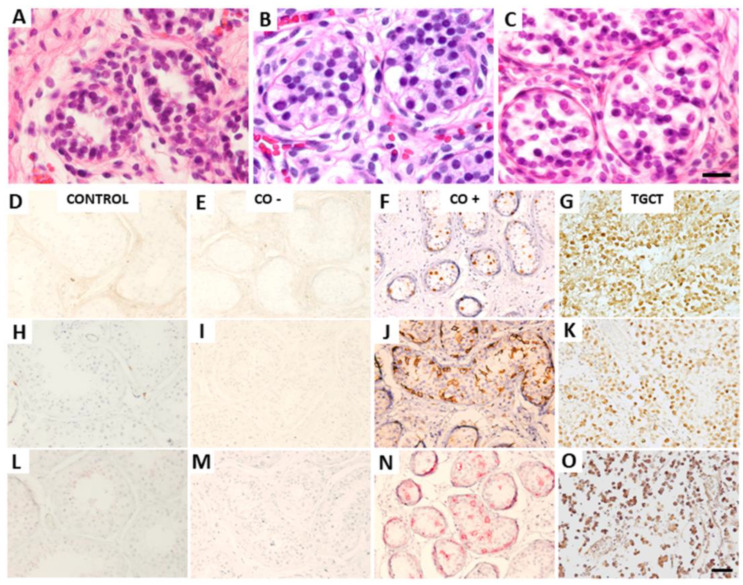
Histological and immunohistochemical analysis. A representative histological comparison among three samples from the CO group after H/E staining is shown at the top. (**A**) Sample CO 9, corresponding to a 10-year-old patient with tubular atrophy. (**B**) Sample CO 4, corresponding to a 12-year-old patient with maturation arrest. (**C**) Sample CO 6, corresponding to 15-year-old patient with tubular atrophy. A representative immunohistochemical comparison of (**D**–**G**) POU5F1, (**H**–**K**) KIT, and (**L**–**O**) PLAP expression is shown at the bottom. From left to right: a sample from Control 1, corresponding to a 17-year-old control individual without immune-positive cells to the antibodies. Sample CO 4, corresponding to a 12-year-old CO patient without immune-positive cells. Sample CO 6, corresponding to a 15-year-old CO patient with immune-positive cells. Sample TGCT 7, corresponding to a 22-year-old TGCT patient with immune-positive cells. CO, cryptorchidism, CO−, CO sample without immune-positive cells, CO+, CO sample with immune-positive cells, TGCT, testicular germ-cell tumors. Calibration bar, 50 μm.

For immunohistochemical analysis of the POU5F1, PLAP, and KIT proteins, no immune-positive cells were observed in the control samples, while they were observed in the TGCT samples, as shown in Table 2. For the CO group, even when some histological alterations were observed, as shown in Figure 1, only 16.7% (2/12) showed immune-positive cells to these proteins, as shown in Table 2 and Figure 1D–F. Therefore, for the CO group, a distinction was made between samples with immune-positive cells (CO+) and those without immuno-positive cells (CO−).

### 3.2. Relative Expression of the hsa-miR-371-373 Cluster and hsa-miR-367

**Figure 2:**
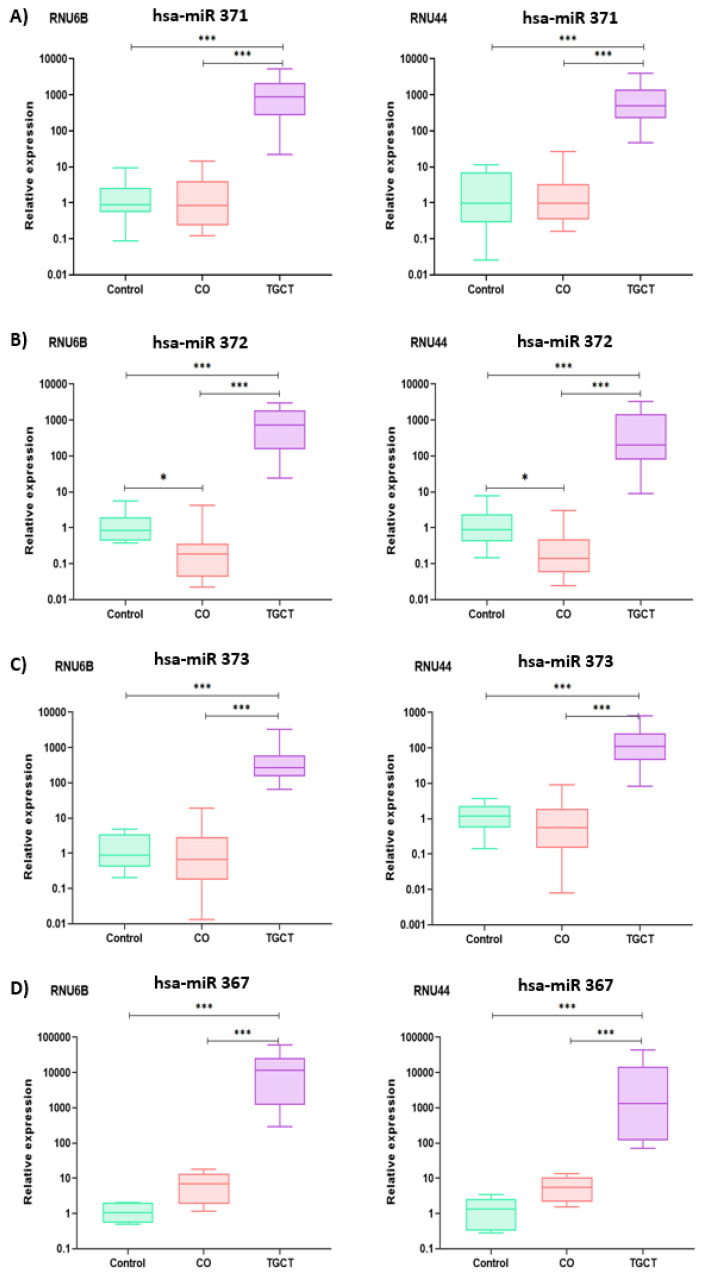
Relative expression graphs of the hsa-miR-371-373 cluster and hsa-miR-367. (**A**) For hsa-miR-371, similar *p* values were observed for both RNU6B and RNU44 (CO vs. control, *p* = ns, CO vs. TGCT, *p* < 0.000, and control vs. TGCT, *p* < 0.000). (**B**) For hsa-miR-372, the following *p* values were observed for RNU6B: CO vs. control, *p* = 0.05, CO vs. TGCT, *p* < 0.000, and control vs. TGCT, *p* < 0.000. For RNU44, we obtained CO vs. control *p* = 0.01, CO vs, TGCT, *p* < 0.000, and control vs. TGCT, *p* < 0.000. (**C**) For hsa-miR-373, similar *p* values were observed for both RNU6B and RNU44 (CO vs. control, *p* = 1.0, CO vs. TGCT, *p* < 0.000, and control vs. TGCT, *p* < 0.000). (**D**) For hsa-miR-367, the following *p* values were observed for RNU6B: CO vs. control, *p* = 0.08, CO vs. TGCT, *p* < 0.000, and control vs. TGCT, *p* = 0.003. For RNU44, we obtained: CO vs. control, *p* = 0.13, CO vs. TGCT, *p* < 0.000, and control vs. TGCT, *p* < 0.000. CO, cryptorchidism, TGCT, testicular germ-cell tumors, ns, not significant, *, *p* < 0.05, ***, *p* < 0.001.

**Figure 3:**
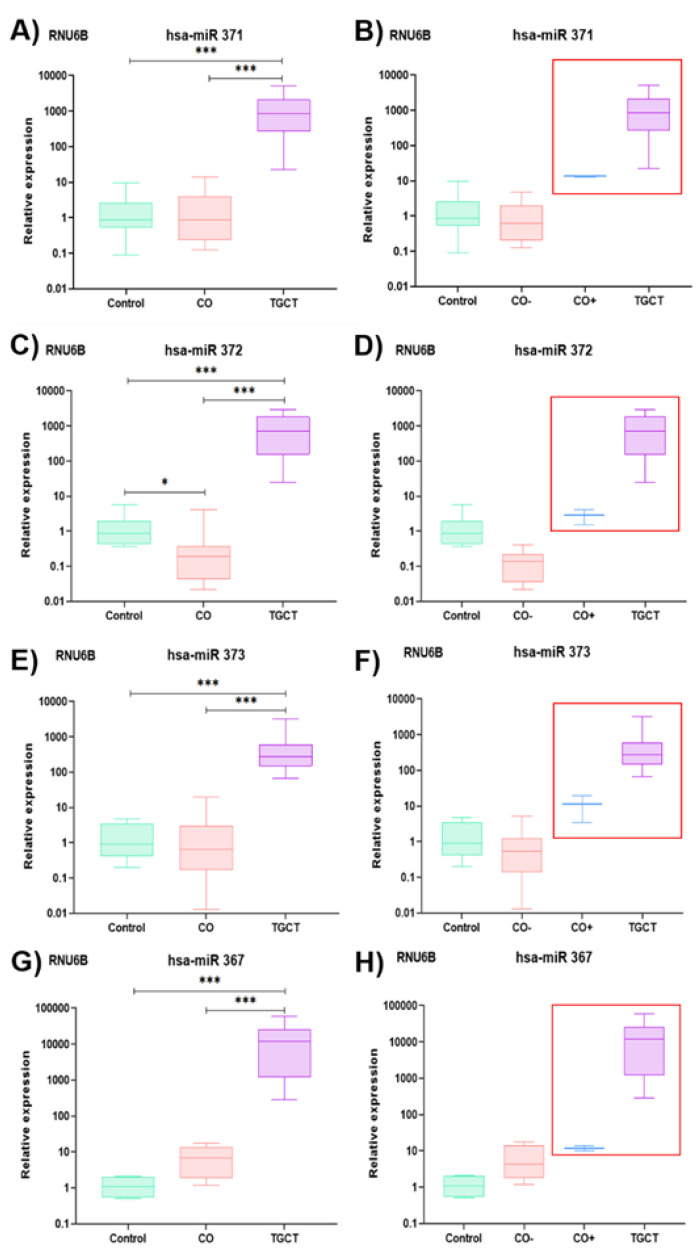
Relative expression graphs of the hsa-miR-371-373 cluster and hsa-miR-367. (**A**) For hsa-miR-371, the following *p* values were observed: CO vs. control, *p* = ns and d of −1.1 (95% confidence interval −5.02 to 2.77), CO vs. TGCT, *p* < 0.000 and d of −1156.61 (95% confidence interval −2082.35 to −230.88), and control vs. TGCT, *p* < 0.000 and d of −1157.74 (95% confidence interval −2243.63 to −71.85); (**B**) corresponding relative expression comparison between the CO− and CO+ subgroups; (**C**) the *p* values for hsa-miR-372 were: CO vs. control, *p* = 0.05 and d of 0.90 (95% confidence interval −0.39 to 2.21), CO vs. TGCT, *p* < 0.000 and d of −913.16 (95% confidence interval −1526.41 to −299.92), and control vs. TGCT, *p* < 0.000 and d of −912.25 (95% confidence interval −1631.59 to −192.92); (**D**) corresponding relative expression comparison between the CO− and CO+ subgroups; (**E**) *p* values for hsa-miR-373: CO vs. control, *p* = ns and d of −1.01 (95% confidence interval −4.94 to 2.92), CO vs. TGCT, *p* < 0.000 and d of −471.29 (95% confidence interval −1008.03 to 65.44), and control vs. TGCT, *p* < 0.000 and d of −472.30 (95% confidence interval −1101.89 to 157.28); (**F**) corresponding relative expression comparison between the CO− and CO+ subgroups; (**G**) *p* values for hsa-miR-367: CO vs. control, *p* = 0.08 and d of −6.74 (95% confidence interval −11.83 to −1.65), CO vs. TGCT, *p* < 0.000 and d of −14,243.28 (95% confidence interval −25,635.86 to −2850.70), and control vs. TGCT, *p* = 0.003 and d of −14,250.03 (95% confidence interval −29,609.78 to 1109.70); (**H**) corresponding relative expression comparison between the CO− and CO+ subgroups. CO, cryptorchidism, CO−, CO sample without immune-positive cells, CO+, CO sample with immune-positive cells, TGCT, testicular germ-cell tumors, ns, not significant, *, *p* < 0.05, ***, *p* < 0.001.

**Figure 4:**
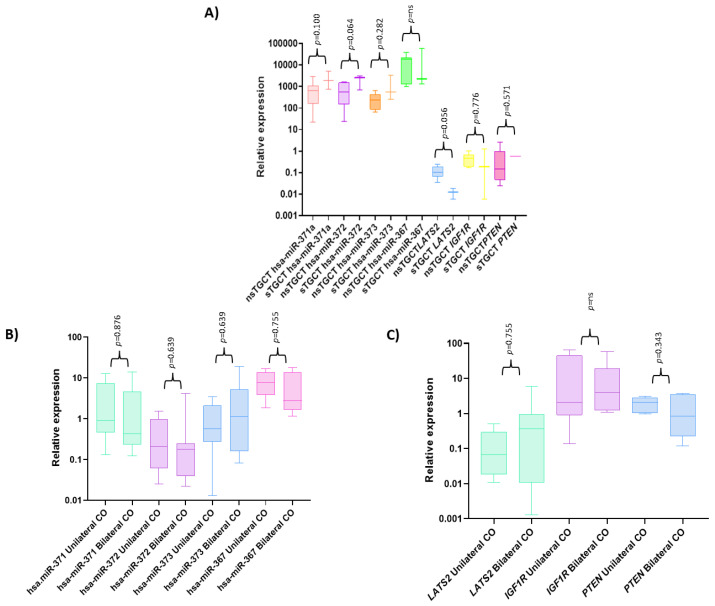
Relative expression graphs of the hsa-miR-371-373 cluster, hsa-miR-367, *LATS2*, *IGF1R*, and *PTEN* according to the samples’ clinical and histological classifications. (**A**) Comparison of gene expression between samples classified as nsTGCT and sTGCT. (**B**) Comparison of the expression of the hsa-miR-371-373 cluster and hsa-miR-367 between samples classified as unilateral CO and bilateral CO. (**C**) Comparison of the expression of *LATS2*, *PTEN*, and *IGF1R* between samples classified as unilateral CO and bilateral CO. CO, cryptorchidism, nsTGCT, non-seminomatous testicular germ-cell tumor, sTGCT, seminomatous testicular germ-cell tumor, ns, not significant.

Regardless of the calibrator used in relative expression determination (RNU44 and RNU6B), consistent behavior was shown, as shown in Figure 2. Therefore, we decided to report only the results normalized according to RNU6B expression. For the TGCT group, the four miRNAs were significantly overexpressed compared with the control and CO samples, as shown in Figure 3A,C,E,G. In contrast, the CO group presented expression levels similar to those of the control group for hsa-miR-371 and hsa-miR-373, as shown in Figure 3A,E. For hsa-miR-372, the CO group presented significantly lower expression levels compared with the other groups, as shown in Figure 3C. Finally, for hsa-miR-367, there were no significant differences in expression levels between the CO and the control groups; however, a tendency to slightly higher values was observed in the CO group, similar to what was observed for the TGCT group, as shown in Figure 3G. However, when the CO samples were divided into a subgroup without immune-positive cells (CO−) and a subgroup with immune-positive cells (CO+) to POU5F1, PLAP, and KIT, the latter showed slightly higher expression levels for the four miRNAs, similar to what was observed for the TGCT group, as shown in Figure 3B,D,F,H. It should be noted that no significant differences were found when comparing the expression of the miRNAs in relation to clinical or pathological characteristics, as shown in Figure 4.

### 3.3. Relative Expression of LATS2, PTEN, and IGF1R

**Figure 5:**
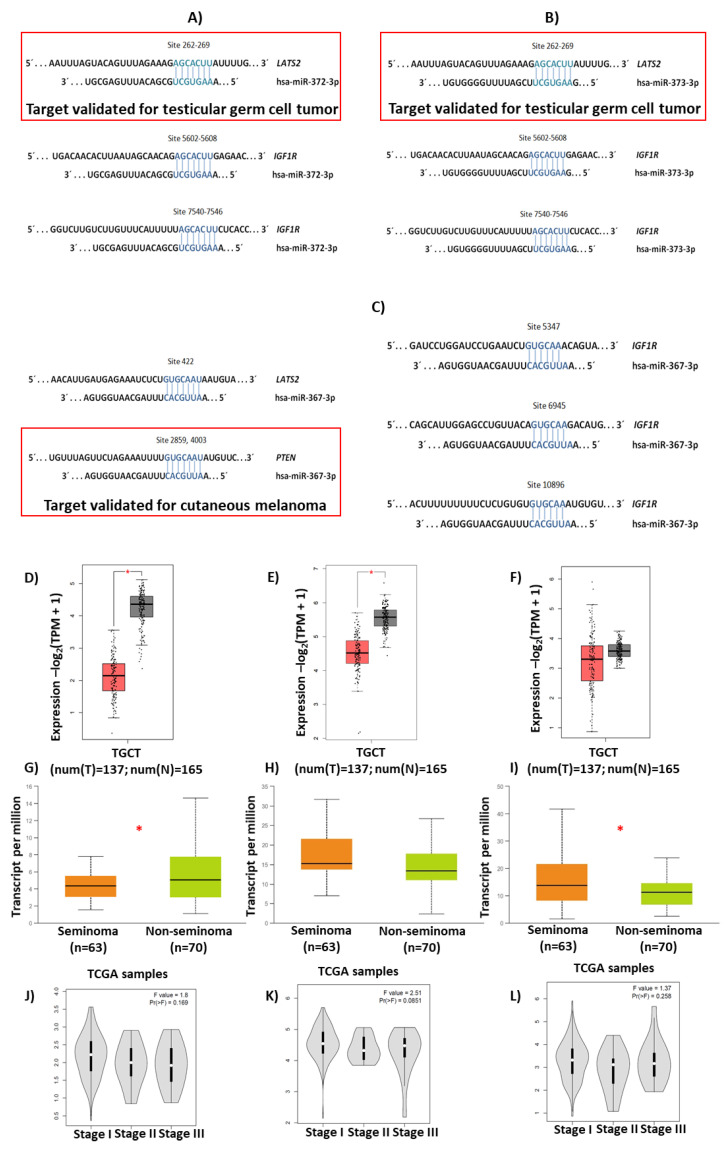
Selection of validated genes. Schematic illustrations of potential binding sites in the 3′-UTR of *LATS2*, *PTEN*, and *IGF1R* for (**A**) hsa-miR-372, (**B**) hsa-miR-373, and (**C**) hsa-miR-367 are shown at the top. In all cases, the validated binding of any miRNA is shown in the red box. Expression of (**D**,**G**,**J**) *LATS2*, (**E**,**H**,**K**) *PTEN*, and (**F**,**I**,**L**) *IGF1R* in tumor tissue compared with healthy tissue, histological classification, and clinical stages of TGCT are shown at the bottom. TCGA, the Cancer Genome Atlas, TGCT, testicular germ-cell tumors, TPM, transcripts per million, *, *p* < 0.001.

**Figure 6:**
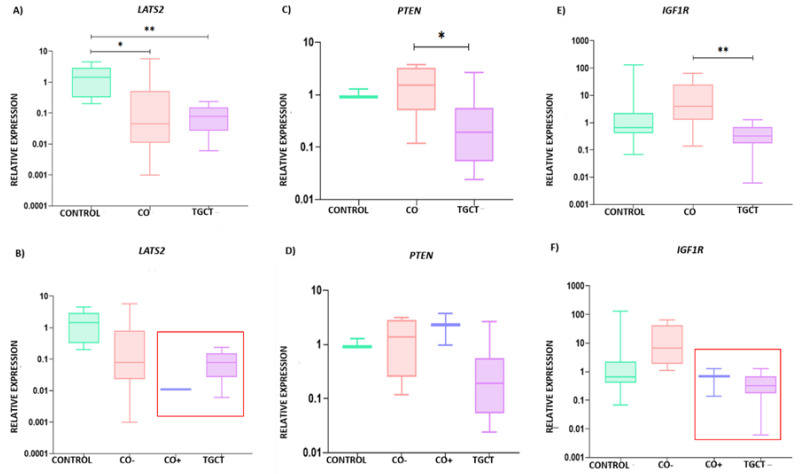
Relative expression graphs of *LATS2*, *PTEN*, e *IGF1R*. (**A**) For *LATS2*, the following *p* values were observed (CO vs. control, *p* = 0.047 and d of 0.91 (95% confidence interval −0.62 to 2.44), control vs. TGCT, *p* < 0.000 and d of 1.55 (95% confidence interval 0.47 to 2.63), and CO vs. TGCT, *p* = ns and d of 0.64 (95% confidence interval −0.63 to 1.93)); (**B**) corresponding relative expression comparison between the CO− and CO+ subgroups; (**C**) *p* values for *PTEN*: CO vs. control, *p* = 1.0 and d of −24.08 (95% confidence interval −57.01 to 8.82), control vs. TGCT, *p* = 0.115 and d of −2.01 (95% confidence interval −7.66 to 3.65), and CO vs. TGCT, *p* = 0.038 and d of 22.08 (95% confidence interval −2.68 to 46.85); (**D**) corresponding relative expression comparison between the CO− and CO+ subgroups; (**E**) *p* values for *IGF1R:* CO vs. control, *p* = 0.074 and d of −0.76 (95% confidence interval −2.81 to 1.28), CO vs. TGCT, *p* < 0.000 and d of 1.20 (95% confidence interval −0.24 to 2.66), and control vs. TGCT, *p* = ns and d of 0.44 (95% confidence interval −0.84 to 1.73); (**F**) corresponding relative expression comparison between the CO− and CO+ subgroups. CO, cryptorchidism, CO−, CO sample without immune-positive cells, CO+, CO sample with immune-positive cells, TGCT, testicular germ-cell tumors, ns, not significant, *, *p* < 0.05, **, *p* < 0.001.

According to the established parameters, we selected the genes *LATS2*, *PTEN*, and *IGF1R* as validated targets of the hsa-miR-371-373 cluster and hsa-miR-367, as shown in Figure 5. In general, the TGCT group showed the lowest expression levels of the three genes analyzed. When comparing the control group to the TGCT group, significant differences were only shown for *LATS2*, as shown in Figure 6A,C,E. Regarding the CO group, it presented significant differences with respect to the control group for the expression of *LATS2*, as shown in Figure 6A. In addition, the CO group presented high expression of *PTEN* and *IGF1R*, which was significantly different when compared with the expression in the TGCT group, as shown in Figure 6C,E. However, when the CO group was divided into the CO− and CO+ subgroups, the latter showed a decrease in *LATS2* and *IGF1R* expression levels, with values similar to those observed for the TGCT group, as shown in Figure 6B,D,F. It should be noted that no significant differences were found when comparing the expressions of *LATS2*, *PTEN*, and *IGF1R* in relation to clinical or pathological characteristics, as shown in Figure 4.

**Figure 7:**
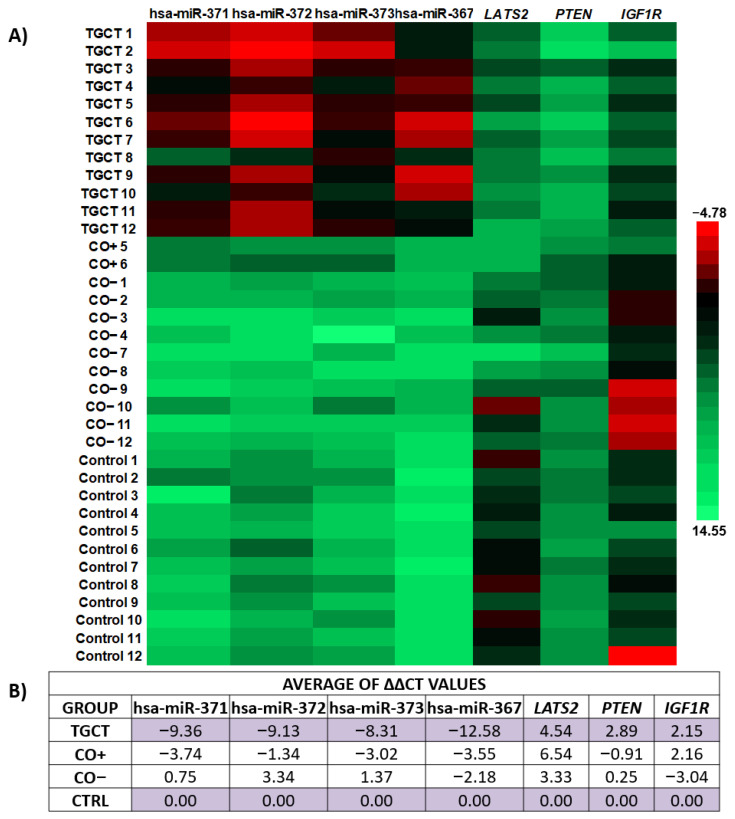
Gene expression levels. (**A**) A heat map is shown with the ∆Ct values for each included sample. The overexpressed genes are shown in red, and the sub-expressed genes are shown in green. The scale is displayed on the side of the heat map. (**B**) Table showing the expression means for each study group expressed in ∆∆Ct. CO−, CO sample without immune-positive cells, CO+, CO sample with immune-positive cells, TGCT, testicular germ-cell tumors.

Due to the clear expression differences between the CO− and CO+ subgroups, we decided to create a heat map with the values of relative expression (∆Ct), in order to show the similarities between the TGCT and the CO+ groups, as shown in Figure 7A. In general, the hsa-miR-371-373 cluster was overexpressed by at least three times in the CO+ subgroup compared with the CO− subgroup, while hsa-miR-367 was overexpressed by 1.3 times in the CO+ subgroup compared with the CO− subgroup. The *LATS2* and *IGF1R* genes were overexpressed by at least three times in the CO+ subgroup compared with the CO- subgroup, as shown in Figure 7B.

### 3.4. Bioinformatic Analysis

**Figure 8:**
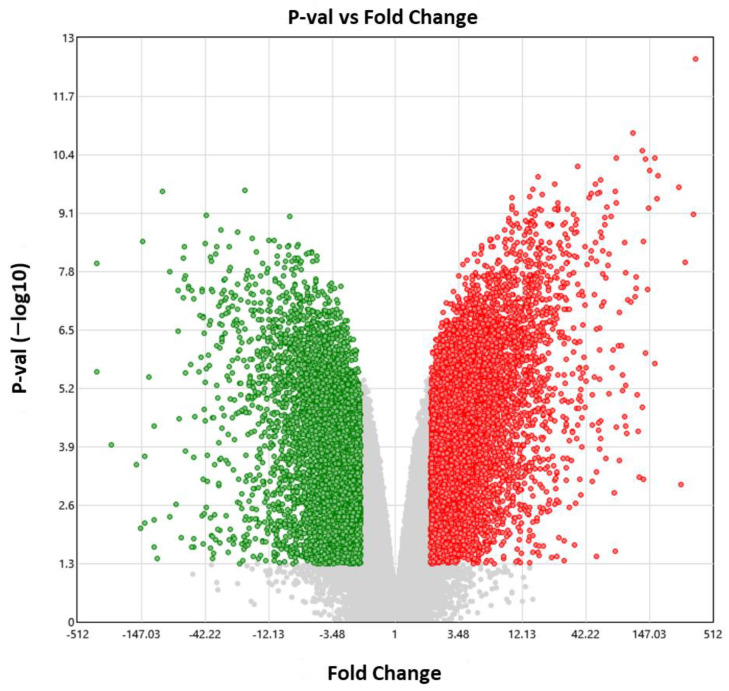
Expression analysis. Volcano plot showing DETs in TGCT. The overexpressed genes are indicated by red dots, while the under-expressed genes are indicated by green dots. DETs, differentially expressed transcripts, TGCT, testicular germ-cell tumors.

**Figure 9:**
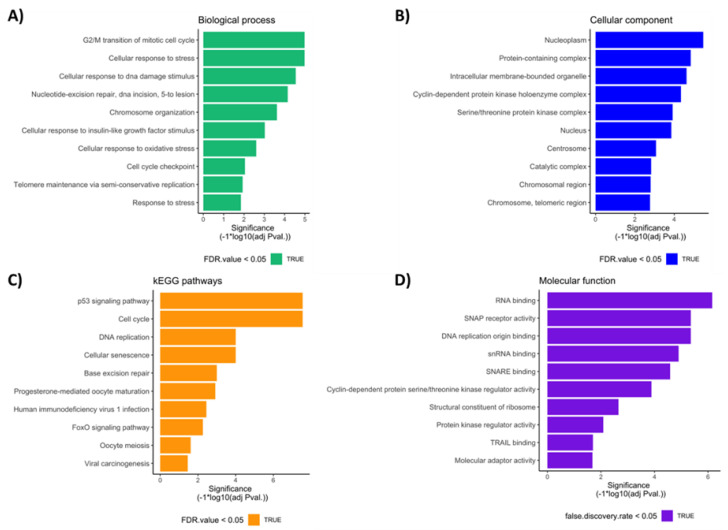
Ontological analysis performed for the under-expressed transcript profile. Bar graphs showing the most important (**A**) biological processes, (**B**) cellular components, (**C**) molecular functions, and (**D**) KEGG pathways associated with the under-expressed transcript profile. FDR value, false discovery rate value, KEGG, Kyoto Encyclopedia of Genes and Genomes.

Using the GEO datasets of the Array Express platform (GSE65026 and GSE25518), 12,858 DETs were visualized in TGCT, of which 7166 transcripts were overexpressed and 5692 transcripts were under-expressed, as shown in Figure 8. Ontological analysis was performed for the under-expressed transcripts, as shown in Figure 9A–D.

**Figure 10:**
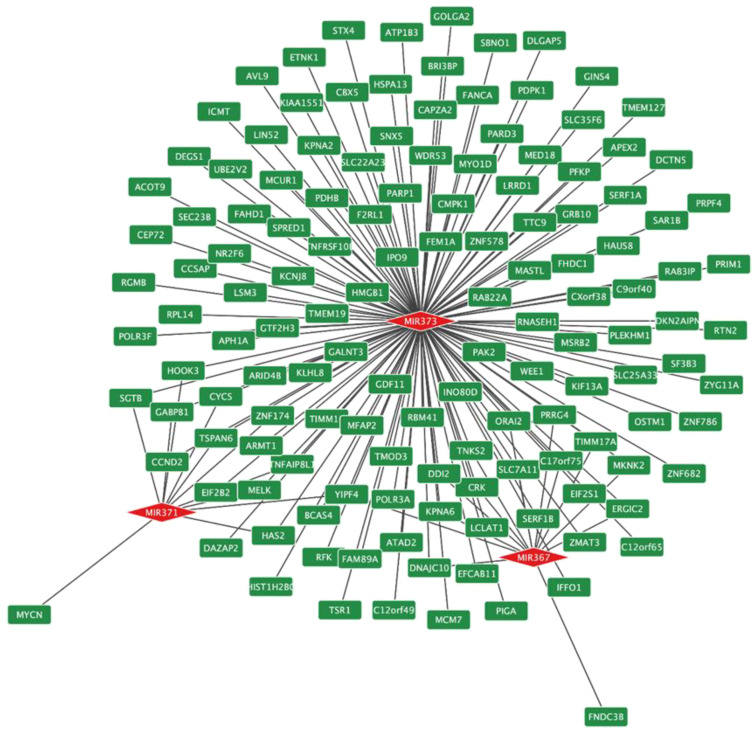
In silico prediction of possible genes co-regulated by the hsa-miR-371-373 cluster and hsa-miR-367. The 143 target genes are shown in green boxes, and the evaluated miRNAs are indicated by red diamonds.

**Figure 11:**
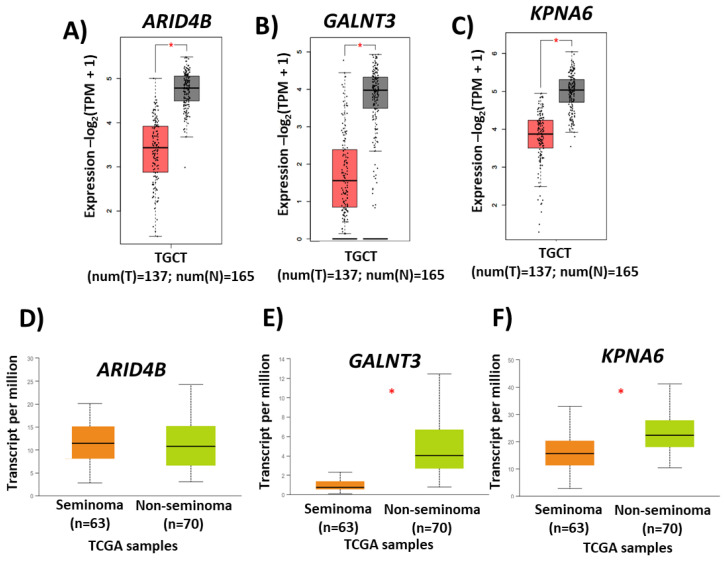
Candidate genes to be validated in the future. The figure shows candidate genes possibly co-regulated by the hsa-miR-371-373 cluster and hsa-miR-367 and differentially expressed in TGCT samples. Comparison of the expression of (**A**,**D**) *ARID4B*, (**B**,**E**) *GALNT3*, and (**C**,**F**) *KPNA6* between TGCT and normal tissue, as well as between seminoma and non-seminoma. TCGA, the Cancer Genome Atlas, TGCT, testicular germ-cell tumors, TPM, transcripts per million, *, *p* < 0.001.

Then, from the under-expression profile, 143 transcripts possibly co-regulated by the hsa-miR-371-373 cluster and hsa-miR-367 were identified, as shown in Figure 10. Finally, among these 143 transcripts, we found differential expression of the AT-rich interaction domain 4B (*ARID4B*), polypeptide N-acetylgalactosaminyltransferase 3 (*GALNT3*), and karyopherin subunit alpha 6 (*KPNA6*) genes in TGCT, as well as in different types of TGCT, as shown in Figure 11. This result supports the validity of our selection of candidate genes to be evaluated in conjunction with *LATS2* and *IGF1R* in a cell model in the future.

## 4. Discussion

CO is the most frequent genitourinary anomaly in male newborns and is one of the most important risk factors for the development of TGCT [1,2]. Although there is a clinical relationship between the two pathologies, the molecular pathways that would suggest an association between the two conditions are unknown [11].

When we integrated our histological and IHC results, we identified tubular atrophy and cells immune-positive for the POU5F1, PLAP, and KIT proteins only in CO+5 (from a patient who was 1 year and 8 months old) and CO+6 (from a 15-year-old patient) samples. By itself, a histological determination is not enough to determine the risk of developing testicular neoplasia; therefore, determining histological parameters in conjunction with the expression of proteins associated with TGCT would allow a comprehensive view of the cell transformation process [23].

In the control and TGCT samples, the expression levels of the hsa-miR-371-373 cluster and hsa-miR-367 were found to be consistent with those described by other authors, even when originating from TGCT patients with a history of CO. The overexpression of these miRNAs was described in serum and tissue samples from patients with TGCT, while they are expressed at basal levels in healthy individuals or in patients’ adjacent testicular tissue [12,15]. In our study, the expression pattern of hsa-miR-371 and hsa-miR-373 in the CO group was similar to that in the control group and showed significant differences only in comparison with that in the TGCT group. hsa-miR-371 is considered the most sensitive and specific miRNA to identify TGCT [14]. This miRNA shows greater diagnostic precision, and its increased expression was associated with relapses and the presentation of metastases, differently from hsa-miR-372, hsa-miR-373 and hsa-miR-367 [24]. Regarding hsa-miR-373, its overexpression was reported during development and in undifferentiated cells [25].

Regarding hsa-miR-372, patients with CO presented a significantly lower expression compared with those with TGCT and the control group. As mentioned above, hsa-miR-372 shares functions with hsa-miR-373 at the testicular level, being expressed in embryonic stem cells and mediating pluripotent capacity in stem cells [26]. Finally, hsa-miR-367 showed intermediate expression in the CO samples with respect to the TGCT and control samples. hsa-miR-367 is part of a cluster that includes hsa-miR-302 and regulates proliferation, differentiation, and the maintenance of pluripotency in embryonic stem cells [27]. Many authors reported the overexpression of hsa-miR-367 in TGCT [12,14]. However, this has not been described in CO.

Interestingly, when evaluating the expression of the four miRNAs in the CO− and CO+ subgroups, we observed that the CO+ subgroup overexpressed them compared with the CO− subgroup and the control group, similar to what was shown for the TGCT group. This finding corroborates the hypothesis that, despite the histological resemblance among CO patients, some of them express characteristic markers of TGCT [28]. In summary, our data showed that the tissues from two CO patients (with CO being a risk factor for the development of TGCT) overexpressed these four miRNAs, similar to what was previously described for hsa-miR-371 in GCNIS samples [29]. In contrast, a recent study identified under-expression of the hsa-miR-371-373 cluster in three patients with CO [30], a result in contrast with ours, possibly due to the low number of patients included in the cited study. Therefore, our results highlight the need to determine the expression of these miRNAs in a larger cohort of patients, with the intention of identifying those CO patients who are at high risk of developing TGCT, as has been proposed in other studies [31,32].

Regarding the relative expression of the *LATS2*, *PTEN*, and *IGF1R* genes, the TGCT group showed low levels of expression, as expected [33,34,35]. Interestingly, we found a significant under-expression of *LATS2* in the CO group compared with the control group, similar to what was observed in the TGCT group. So far, no reports have been published on the expression of the *LATS2* gene in CO. It was reported that the overexpression of hsa-miR-372 and hsa-miR-373 in TGCT was associated with a reduced expression of the LATS2 protein. In turn, it was shown that the under-expression of *LATS2* induced a high expression of CDK2, promoting the transition of cancer cells from the G1 to the S phase of the cell cycle [36]. Therefore, it is proposed that this regulation could occur in patients with CO at risk of developing a testicular malignancy.

Regarding *PTEN* and *IGF1R*, these genes were overexpressed in the CO group, with a significant difference with respect to the TGCT group. To date, there are no reports on the expression of these two genes in CO. In melanoma, hsa-miR-367 inhibits the expression of the *PTEN* gene, increasing the growth and invasion of cancer cells, while, in hepatocarcinoma, hsa-miR-371 inhibits *PTEN* gene expression, promoting the proliferation and metastasis of cancer cells [37,38]. This gene is under-expressed in seminoma, embryonal carcinoma, and teratoma [34]. Low levels of *PTEN* were also related to DNA damage repair and the maintenance of genomic integrity [39]. However, based on our results, we did not observe a clear relationship between this gene and the evaluated miRNAs in the CO patients, evidencing that CO patients positive for malignancy markers do not present alterations in this regulatory pathway. In the case of *IGF1R*, differences in gene expression between seminomatous and non-seminomatous TGCT were described [40], and, through our in silico analyses, we identified it as a gene possibly regulated by hsa-miR-372-373 and hsa-miR-367; however, there are no reports that validate the regulation of this gene by the aforementioned miRNAs. *IGF1R* participates in the development of the cremaster muscle, testicular descent, adrenogenital development, and testicular maturation, as well as in the proliferation, cell survival, and differentiation of Sertoli cells and germ cells [41]. Therefore, dysfunction in the processes regulated by this gene could equally be present in CO patients at risk of TGCT development.

Once again, when comparing the expression of the three mentioned genes between the CO− and CO+ subgroups, we observed that the CO+ subgroup under-expressed the *LAST2* and *IGF1R* genes, similar to what was shown for the TGCT group compared with the CO− subgroup and the control group. Therefore, we do not rule out the existence of possible regulatory pathways involving both genes.

When performing a global summary of the results obtained for the CO+ subgroup (2/12), we observed overexpression of the hsa-miR-371-373 cluster as well as of hsa-miR-367 and under-expression of *LATS2* and *IGF1R*. Previously, it was reported that 5.7% of patients with CO express the proteins POU5F1, PLAP, SALL4, AP2γ, and KIT, which are associated with the risk of developing GCNIS, the precursor lesion of TGCT [6,23,28], This is consistent with our results, indicating that 16.7% of the CO patients had these characteristics. In addition, it was shown that, both in gonocytes and in TGCT, the hsa-miR-302-367 clusters are regulated by transcription factors such as POU5F1, NANOG, and SOX2 [42] and that they are co-expressed with proliferation-associated proteins such as KIT and even specific proteins of germ cells such as PLAP [6].

Finally, the in silico analysis of expression we carried out allowed us to identify *ARID4B,* which acts as a coactivator of the androgen receptor and is expressed in Sertoli cells, which participate in the development of germ cells and in the formation of the blood–testicular barrier [43,44]; *GALNT3,* which is overexpressed in spermatocytes and spermatids from healthy individuals and has been described in testicular microlithiasis [45,46] (it is pertinent to reiterate that testicular microlithiasis, by itself, does not represent a malignant condition, but when associated with other conditions as CO, can be considered a risk factor for the development of TGCT [47]); and *KPNA6,* which is expressed in the testicle and, when its expression is inhibited, affects spermatogenesis, causing infertility [48] as other possible miRNA target genes, susceptible to future evaluation together with the hsa-miR-371-373 cluster, hsa-miR-367, *LATS2* and *IGF1R*. Integrating a systemic phenomics approach will allow us to study these genetic markers in a comprehensive manner, complementing these data with more samples from clinical files and in vivo or in vitro assays, and even including some other biochemical determinations to deepen their suitability as diagnostic markers [49].

This is the first preliminary report that describes how the testicular tissues from two CO patients with cells immune-positive to POU5F1, PLAP, and KIT overexpressed the hsa-miR-371-373 cluster and hsa-miR-367 and under-expressed the *LATS2* and *IGF1R* genes, similar to what was observed in TGCT tissues. We must state that our study shows results of a preliminary nature, and, thus, it has several limitations that must also be considered in relation to future works. Undoubtedly, the main limitation is the feasibility of obtaining testicular tissue from CO patients, since routinely taking testicular biopsies is not indicated in clinical practice. For this reason, not many studies analyzed this type of sample with the intention of identifying molecular markers related to TGCT. The accessibility of these archival samples is limited, which had a direct impact on the number of expression assays we carried out. Therefore, in the future it would be important to determine the expression of the *ARID4B*, *GALNT3*, and *KPNA6* genes, which could not be done in this study. Another limitation derived from the little amount of tissue available was our insufficient ability to determine gene expression in situ after microdissection of the gonocytes from these tissues. Being able to make this determination will be necessary to corroborate the hypothesis that these markers are exclusively expressed in the gonocytes, which would support the theory according to which the gonocytes are the target of malignancy mechanisms leading to the development of TGCT in CO patients. Taking these weaknesses into account, we think that, in the future, it will be important to replicate the present study with a larger cohort of patients, since at the moment our preliminary study included only 12 samples from CO patients. In addition, it will be necessary to evaluate in a cell model whether the *IGF1R*, *ARID4B*, *GALNT3*, and *KPNA6* genes are indeed targets of the examined miRNAs. We believe that, if these results are confirmed, the determination of the expression of these miRNAs could be very useful in clinical practice in the future; specifically, it would help to distinguish those pediatric CO patients with a higher risk of developing TGCT in adulthood. However, due to the nature of our results, this statement is speculative.

## 5. Conclusions

This is the first report that, through genetic expression data, supports the theory according to which gonocytes are a target of malignancy processes leading to the development of TGCT in pediatric CO patients. However, it should be noted that these results are preliminary and need to be validated in a larger cohort of patients.

## Figures and Tables

**Table 1:** Clinical, histological, and pathological characteristics of the samples. (**A**) Control group. (**B**) CO group. (**C**) TGCT group. nsTGCT, non-seminomatous testicular germ-cell tumor, sTGCT, seminomatous testicular germ-cell tumor, S, seminoma, T, teratoma, YS, yolk-sack tumor, CH, choriocarcinoma, CO, cryptorchidism, TGCT, testicular germ-cell tumors.

**(A) Control group**								
**Case**			**Age**				**Histology**	
Control 1			17 years				Normal spermatogenesis	
Control 2			8 years				Normal for age	
Control 3			39 years				Normal spermatogenesis	
Control 4			17 years				Normal spermatogenesis	
Control 5			17 years				Hypospermatogenesis	
Control 6			18 years				Normal spermatogenesis	
Control 7			18 years				Normal spermatogenesis	
Control 8			18 years				Normal spermatogenesis	
Control 9			22 years				Normal spermatogenesis	
Control 10			4 years				Normal for age	
Control 11			1 year				Normal for age	
Control 12			14 years				Normal spermatogenesis	
**(B) CO group**								
**Case**		**Age**	**Personal history of CO**		**CO laterality**		**Histology**	
CO 1		1 year	Non-syndromic		Bilateral		Normal for age	
CO 2		2 years	Non-syndromic		Right		Tubular atrophy	
CO 3		1 years	Non-syndromic		Left		Normal for age	
CO 4		12 years	Non-syndromic		Bilateral		Maturation arrest	
CO 5		1 years	Non-syndromic		Bilateral		Tubular atrophy	
CO 6		15 years	Non-syndromic		Right		Tubular atrophy	
CO 7		1 year	Non-syndromic		Left		Normal for age	
CO 8		4 years	Non-syndromic		Left		Normal for age	
CO 9		10 years	Non-syndromic		Bilateral		Tubular atrophy	
CO 10		5 years	Non-syndromic		Left		Tubular atrophy	
CO 11		1 year	Non-syndromic		Left		Tubular atrophy	
CO 12		8 years	Non-syndromic		Bilateral		Tubular atrophy	
**(C) TGCT group**								
**Case**	**Age**	**Diagnosis**	**Histology**	**TGCT laterality**	**Clinical stage**	**Metastasis**	**Personal history of CO**	**CO laterality**
TGCT 1	49 years	nsTGCT	T	Right	ll	No	Non-syndromic	Bilateral
TGCT 2	27 years	sTGCT	S	Right	I	No	Non-syndromic	Bilateral
TGCT 3	17 years	nsTGCT	T	Left	II	Pelvis	Non-syndromic	Bilateral
TGCT 4	19 years	nsTGCT	YS + CH	Left	ll	Retroperitoneum	Non-syndromic	Left
TGCT 5	16 years	nsTGCT	T	Right	I	No	Non-syndromic	Bilateral
TGCT 6	22 years	sTGCT	S	Left	II	Retroperitoneum	Non-syndromic	Left
TGCT 7	22 years	nsTGCT	S + T	Bilateral	II	No	Non-syndromic	Bilateral
TGCT 8	16 years	nsTGCT	S + T + CH	Right	II	Retroperitoneum	Non-syndromic	Bilateral
TGCT 9	23 years	nsTGCT	T	Right	IV	Lung	Non-syndromic	Right
TGCT 10	27 years	nsTGCT	T	Left	ll	Retroperitoneum	Non-syndromic	Left
TGCT 11	18 years	sTGCT	S	Right	l	No	Non-syndromic	Right
TGCT 12	24 years	nsTGCT	S +T	Right	l	No	Non-syndromic	Right

**Table 2:** Immunopositivity to POU5F1, PLAP, and KIT proteins in the samples.

Sample	Control			CO			TGCT		
	POU5F1	PLAP	KIT	POU5F1	PLAP	KIT	POU5F1	PLAP	KIT
1	−	−	−	−	−	−	+	−	−
2	−	−	−	−	−	−	+	+	+
3	−	−	−	−	−	−	−	−	−
4	−	−	−	−	−	−	−	+	+
5	−	−	−	+	+	+	−	−	−
6	−	−	−	+	+	+	+	+	+
7	−	−	−	−	−	−	+	+	+
8	−	−	−	−	−	−	+	+	+
9	−	−	−	−	−	−	−	−	−
10	−	−	−	−	−	−	+	−	−
11	−	−	−	−	−	−	+	+	+
12	−	−	−	−	−	−	+	+	+

+, immuno-positive, −, not immuno-positive, CO, cryptorchidism, TGCT, testicular germ cell tumors.

## Data Availability

Not applicable.

## Supplementary Materials

The following supporting information can be downloaded at: https://www.mdpi.com/article/10.3390/diagnostics13183020/s1, Figure S1: RT-qPCR conditions. General conditions of RT-qPCR assays. (A) microRNA probes evaluated in our study. (B) mRNA probes evaluated in our study. (C) RT-PCR conditions used in our study. (D) qPCR conditions used in our study.

## References

[1] Elamo H.P., Virtanen H.E., Toppari J. (2022). Genetics of cryptorchidism and testicular regression. Best Pr. Res. Clin. Endocrinol. Metab..

[2] Rodprasert W., Virtanen H.E., Mäkelä J.-A., Toppari J. (2020). Hypogonadism and Cryptorchidism. Front. Endocrinol..

[3] Skakkebæk N.E., Berthelsen J.G., Giwercman A., Müller J. (1987). Carcinoma-in-situ of the testis: Possible origin from gonocytes and precursor of all types of germ cell tumours except spermatocytoma. Int. J. Androl..

[4] Oosterhuis J.W., Looijenga L.H.J. (2005). Testicular germ-cell tumours in a broader perspective. Nat. Rev. Cancer.

[5] Gillis A.J.M., Stoop H., Biermann K., van Gurp R.J.H.L.M., Swartzman E., Cribbes S., Ferlinz A., Shannon M., Oosterhuis J., Looijenga L.H.J. (2011). Expression and interdependencies of pluripotency factors LIN28, OCT3/4, NANOG and SOX2 in human testicular germ cells and tumours of the testis. Int. J. Androl..

[6] Vigueras-Villaseñor R.M., Cortés-Trujillo L., Chávez-Saldaña M., Vázquez F.G., Carrasco-Daza D., Cuevas-Alpuche O., Rojas-Castañeda J.C. (2015). Analysis of POU5F1, c-Kit, PLAP, AP2γ and SALL4 in gonocytes of patients with cryptorchidism. Acta Histochem..

[7] Tien M.Y., Abeydeera S.A., Cho H.-J., Sarila G., Catubig A., Burton E., Hutson J., Li R. (2020). Does the apoptosis pathway play a critical role in gonocyte transformation?. J. Pediatr. Surg..

[8] Hutson J.M., Li R., Southwell B.R., Petersen B.L., Thorup J., Cortes D. (2013). Germ cell development in the postnatal testis: The key to prevent malignancy in cryptorchidism?. Front. Endocrinol..

[9] Novotny G.W., Belling K.C., Bramsen J.B., E Nielsen J., Bork-Jensen J., Almstrup K., Sonne S.B., Kjems J., Meyts E.R.-D., Leffers H. (2012). MicroRNA expression profiling of carcinoma in situ cells of the testis. Endocrine-Related Cancer.

[10] Radtke A., Cremers J.-F., Kliesch S., Riek S., Junker K., Mohamed S.A., Anheuser P., Belge G., Dieckmann K.-P. (2017). Can germ cell neoplasia in situ be diagnosed by measuring serum levels of microRNA371a-3p?. J. Cancer Res. Clin. Oncol..

[11] García-Andrade F., Vigueras-Villaseñor R.M., Chávez-Saldaña M.D., Rojas-Castañeda J.C., Bahena-Ocampo I.U., Aréchaga-Ocampo E., Díaz-Chávez J., Landero-Huerta D.A. (2022). The Role of microRNAs in the Gonocyte Theory as Target of Malignancy: Looking for Potential Diagnostic Biomarkers. Int. J. Mol. Sci..

[12] Gillis A.J., Rijlaarsdam M.A., Eini R., Dorssers L.C., Biermann K., Murray M.J., Nicholson J.C., Coleman N., Dieckmann K.-P., Belge G. (2013). Targeted serum miRNA (TSmiR) test for diagnosis and follow-up of (testicular) germ cell cancer patients: A proof of principle. Mol. Oncol..

[13] Regouc M., Belge G., Lorch A., Dieckmann K.-P., Pichler M. (2020). Non-Coding microRNAs as Novel Potential Tumor Markers in Testicular Cancer. Cancers.

[14] van Agthoven T., Looijenga L.H. (2016). Accurate primary germ cell cancer diagnosis using serum based microRNA detection (ampTSmiR test). Oncotarget.

[15] Palmer R.D., Murray M.J., Saini H.K., van Dongen S., Abreu-Goodger C., Muralidhar B., Pett M.R., Thornton C.M., Nicholson J.C., Enright A.J. (2010). Malignant Germ Cell Tumors Display Common MicroRNA Profiles Resulting in Global Changes in Expression of Messenger RNA Targets. Cancer Res.

[16] Chang Y., Wang X., Xu Y., Yang L., Qian Q., Ju S., Chen Y., Chen S., Qin N., Ma Z. (2019). Comprehensive characterization of cancer-testis genes in testicular germ cell tumor. Cancer Med..

[17] Chen Y., Wang X. (2019). miRDB: An online database for prediction of functional microRNA targets. Nucleic Acids Res..

[18] Agarwal V., Bell G.W., Nam J.-W., Bartel D.P. (2015). Predicting effective microRNA target sites in mammalian mRNAs. eLife.

[19] Li C., Tang Z., Zhang W., Ye Z., Liu F. (2021). GEPIA2021: Integrating multiple deconvolution-based analysis into GEPIA. Nucleic Acids Res..

[20] Chandrashekar D.S., Bashel B., Balasubramanya S.A.H., Creighton C.J., Ponce-Rodriguez I., Chakravarthi B.V.S.K., Varambally S. (2017). UALCAN: A portal for facilitating tumor subgroup gene expression and survival analyses. Neoplasia.

[21] Mi H., Muruganujan A., Ebert D., Huang X., Thomas P.D. (2018). PANTHER version 14: More genomes, a new PANTHER GO-slim and improvements in enrichment analysis tools. Nucleic Acids Res..

[22] Kanehisa M., Goto S. (2000). KEGG: Kyoto Encyclopedia of Genes and Genomes. Nucleic Acids Res..

[23] Kvist K., Clasen-Linde E., Langballe O., Hansen S.H., Cortes D., Thorup J. (2018). The Expression of Markers for Intratubular Germ Cell Neoplasia in Normal Infantile Testes. Front. Endocrinol..

[24] Murray M.J., Bell E., Raby K.L., A Rijlaarsdam M., Gillis A.J.M., Looijenga L.H.J., Brown H., Destenaves B., Nicholson J.C., Coleman N. (2015). A pipeline to quantify serum and cerebrospinal fluid microRNAs for diagnosis and detection of relapse in paediatric malignant germ-cell tumours. Br. J. Cancer.

[25] Rosa A., Papaioannou M.D., Krzyspiak J.E., Brivanlou A.H. (2014). miR-373 is regulated by TGFβ signaling and promotes mesendoderm differentiation in human Embryonic Stem Cells. Dev. Biol..

[26] Stadler B., Ivanovska I., Mehta K., Song S., Nelson A., Tan Y., Mathieu J., Darby C., Blau C.A., Ware G. (2010). Characterization of microRNAs Involved in Embryonic Stem Cell States. Stem Cells Dev..

[27] Guo M., Gan L., Si J., Zhang J., Liu Z., Zhao J., Gou Z., Zhang H. (2020). Role of miR-302/367 cluster in human physiology and pathophysiology. Acta Biochim. Biophys. Sin..

[28] Clasen-Linde E., Kvist K., Cortes D., Thorup J. (2015). The value of positive Oct3/4 and D2-40 immunohistochemical expression in prediction of germ cell neoplasia in prepubertal boys with cryptorchidism. Scand. J. Urol..

[29] Lobo J., van Zogchel L.M.J., Nuru M.G., Gillis A.J.M., van der Schoot C.E., Tytgat G.A.M., Looijenga L.H.J. (2021). Combining Hypermethylated *RASSF1A* Detection Using ddPCR with miR-371a-3p Testing: An Improved Panel of Liquid Biopsy Biomarkers for Testicular Germ Cell Tumor Patients. Cancers.

[30] Tang D., Huang Z., He X., Wu H., Peng D., Zhang L., Zhang X. (2018). Altered miRNA profile in testis of post-cryptorchidopexy patients with non-obstructive azoospermia. Reprod. Biol. Endocrinol..

[31] Woo C.G., Lee O.-J., Yang Y., Kim Y.J., Lee J., Son S.-M. (2019). Collision tumor comprising metastatic cholangiocarcinoma and seminoma in an undescended testis: A case report. J. Int. Med Res..

[32] Osterballe L., Clasen-Linde E., Cortes D., Engholm G., Hertzum-Larsen R., Reinhardt S., Thorup J. (2017). The diagnostic impact of testicular biopsies for intratubular germ cell neoplasia in cryptorchid boys and the subsequent risk of testicular cancer in men with prepubertal surgery for syndromic or non-syndromic cryptorchidism. J. Pediatr. Surg..

[33] Duale N., Lindeman B., Komada M., Olsen A.-K., Andreassen A., Soderlund E.J., Brunborg G. (2007). Molecular portrait of cisplatin induced response in human testis cancer cell lines based on gene expression profiles. Mol. Cancer.

[34] Yang N.-Q., Luo X.-J., Zhang J., Wang G.-M., Guo J.-M. (2016). Crosstalk between Meg3 and miR-1297 regulates growth of testicular germ cell tumor through PTEN/PI3K/AKT pathway. Am. J. Transl. Res..

[35] Selfe J., Shipley J.M. (2019). IGFsignalling in germ cells and testicular germ cell tumours: Roles and therapeutic approaches. Andrology.

[36] Voorhoeve P.M., le Sage C., Schrier M., Gillis A.J., Stoop H., Nagel R., Liu Y.-P., van Duijse J., Drost J., Griekspoor A. (2006). A Genetic Screen Implicates miRNA-372 and miRNA-373 As Oncogenes in Testicular Germ Cell Tumors. Cell.

[37] Long J., Luo J., Yin X. (2018). miR-367 enhances the proliferation and invasion of cutaneous malignant melanoma by regulating phosphatase and tensin homolog expression. Mol. Med. Rep..

[38] Wang H., Zhao Y., Chen T., Liu G., He N., Hu H. (2019). MiR-371 promotes proliferation and metastasis in hepatocellular carcinoma by targeting PTEN. BMB Rep..

[39] Shin J.W., Kim S.-H., Yoon J.Y. (2021). PTEN downregulation induces apoptosis and cell cycle arrest in uterine cervical cancer cells. Exp. Ther. Med..

[40] Neuvians T.P., Gashaw I., Hasenfus A., Häcker A., Winterhager E., Grobholz R. (2005). Differential Expression of IGF Components and Insulin Receptor Isoforms in Human Seminoma Versus Normal Testicular Tissue. Neoplasia.

[41] Cannarella R., Condorelli R.A., La Vignera S., Calogero A.E. (2017). Effects of the insulin-like growth factor system on testicular differentiation and function: A review of the literature. Andrology.

[42] Liu J., Wang Y., Ji P., Jin X. (2020). Application of the microRNA-302/367 cluster in cancer therapy. Cancer Sci..

[43] Wu R.-C., Jiang M., Beaudet A.L., Wu M.-Y. (2013). ARID4A and ARID4B regulate male fertility, a functional link to the AR and RB pathways. Proc. Natl. Acad. Sci. USA.

[44] Wu R.-C., Zeng Y., Pan I.-W., Wu M.-Y. (2015). Androgen Receptor Coactivator ARID4B Is Required for the Function of Sertoli Cells in Spermatogenesis. Mol. Endocrinol..

[45] Miyazaki T., Mori M., Yoshida C.A., Ito C., Yamatoya K., Moriishi T., Kawai Y., Komori H., Kawane T., Izumi S.-I. (2012). Galnt3 deficiency disrupts acrosome formation and leads to oligoasthenoteratozoospermia. Histochem..

[46] Campagnoli M.F. (2006). Familial tumoral calcinosis and testicular microlithiasis associated with a new mutation of GALNT3 in a white family. J. Clin. Pathol..

[47] Richenberg J., Belfield J., Ramchandani P., Rocher L., Freeman S., Tsili A.C., Cuthbert F., Studniarek M., Bertolotto M., Turgut A.T. (2014). Testicular microlithiasis imaging and follow-up: Guidelines of the ESUR scrotal imaging subcommittee. Eur. Radiol..

[48] Liu N., Qadri F., Busch H., Huegel S., Sihn G., Chuykin I., Hartmann E., Bader M., Rother F. (2021). Kpna6 deficiency causes infertility in male mice by disrupting spermatogenesis. Development.

[49] Ying W. (2023). Phenomic Studies on Diseases: Potential and Challenges. Phenomics.

