# Supplementary material for: Molecular Characterization of Patients with Cryptorchidism: Preliminary Search for an Expression Profile Related to That of Testicular Germ-Cell Tumors
Source: Diagnostics (Basel). 2023 Sep 21;13(18):3020. doi: 10.3390/diagnostics13183020 (PMC10529510; doi:10.3390/diagnostics13183020)
Supplement: Supplementary file 1 [file diagnostics-13-03020-s001.zip › diagnostics-2476921-supplementary.pdf]

A) microRNA probes

miRNA	ID	Sequence from 5' to 3'
hsa-miR-371a-3p	002124	AAGUGCCGCCAUCUUUUGAGUGU
hsa-miR-372-3p	000560	AAAGUGCUGCGACAUUUGAGCGU
hsa-miR-373-3p	000561	GAAGUGCUUCGAUUUUGGGGUGU
hsa-miR-367-3p	000555	AAUUGCACUUUAGCAAUGGUGA
RNU6B	001093	CGCAAGGATGACACGCAAATTCGTGAAGCGTTCCATATTTTT
RNU44	001094	CCTGGATGATGATAGCAAATGCTGACTGAACATGAAGGTCTT AATTAGCTCTAACTGACT

B) mRNA probes

Gen	ID	Amplicon length
PTEN	Hs008229813_s1	154
LATS2	Hs1059009_m1	68
IGF1R	Hs01075031_s1	98
GAPDH	Hs04420566_g1	120

C) RT-PCR Conditions

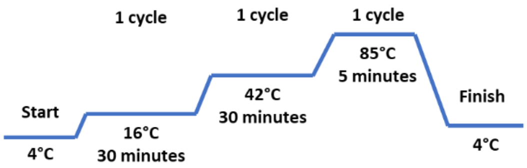

D) qPCR Conditions

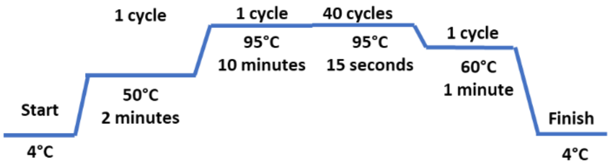

Supplementary Figure 1. RT-qPCR conditions. General conditions of RT-qPCR assays. **A)** microRNA probes evaluated in our study. **B)** mRNA probes evaluated in our study. **C)** RT-PCR conditions used in our study. **D)** qPCR conditions used in our study.
